# Exploring the Physiological and Psychological Effects of Digital Shinrin-Yoku and Its Characteristics as a Restorative Environment

**DOI:** 10.3390/ijerph19031202

**Published:** 2022-01-21

**Authors:** Norimasa Takayama, Takeshi Morikawa, Kazuko Koga, Yoichi Miyazaki, Kenichi Harada, Keiko Fukumoto, Yuji Tsujiki

**Affiliations:** 1 Department of Forest Management, Forestry and Forest Products Research Institute, 1 Matsunosato, Tsukuba 305-8687, Ibaraki, Japan; 2 Department of Wood Engineering, Forestry and Forest Products Research Institute, 1 Matunosato, Tsukuba 305-8687, Ibaraki, Japan; tmorik@ffpri.affrc.go.jp; 3 Independent Researcher, 5 Yatsu, Narashino 275-0026, Chiba, Japan; kazukoga923@gmail.com; 4 Forestdigital Inc., 1-51 Tokomuro, Urahoro, Tokachi 089-5633, Hokkaido, Japan; yoichi.miyazaki@forestdigital.org (Y.M.); kenichi.harada@forestdigital.org (K.H.); yuji.tsujiki@forestdigital.org (Y.T.); 5 Shikoku Research Center, Forestry and Forest Products Research Institute, 2-915 Asakuranishi, Kochi 780-8077, Kochi, Japan; kfukumoto@ffpri.affrc.go.jp

**Keywords:** digital Shinrin-yoku, forest bathing, physiological effect, psychological effect, restorative trait, virtual experience, visual element, auditory element, olfactory element

## Abstract

This study investigated the physiological and psychological therapeutic effects of a digital Shinrin-yoku environment constructed indoors in an urban facility as well as the characteristics of the environment that contribute to restorativeness (restorative traits). We measured the fluctuations in the physical and mental states of 25 subjects by obtaining both before–after measurements and continuous measurements while exposed to a digital Shinrin-yoku environment that reproduced visual, auditory, and olfactory elements. The results demonstrated that the parasympathetic nerve activity was significantly increased and that the heart rate was significantly decreased during the exposure compared with that during the resting state. As for mood, five of the six Profile of Mood States (POMS) scales (“Tension–Anxiety,” “Depression,” “Anger–Hostility,” “Fatigue,” and “Confusion”) were significantly decreased after the experience. In addition, psychological restorative effects were also confirmed, with a significant decrease in “negative affect” (measured using the Positive and Negative Affect Schedule (PANAS)) and a significant increase in the sense of restorativeness (Restorative Outcome Scale (ROS)) after the experience. In contrast, comparing the digital Shinrin-yoku environment with the actual forest environment and the urban environment using POMS, PANAS, ROS, and Perceived Restorativeness Scale (PRS), the psychological effects and environmental traits of the digital Shinrin-yoku were found to be considerably similar to those of the actual forest environment.

## 1. Introduction

There have been many studies on indoor and outdoor forest bathing [1,2,3,4,5,6,7,8,9,10]. In recent years, many review articles have focused on specific measurement indices [11,12,13,14], with some articles providing a reasonably high level of evidence [15,16,17]. Most studies have focused on the effects of nature and forests on physical health, including on improvements in physiological parameters such as blood pressure, autonomic nervous system, and various hormones. Recently, however, research on improving psychological health, such as depression [18,19] and trauma [20], has also increased.

Unfortunately, in the past 2 years, the world has been hit by a pandemic due to a new coronavirus infection. As a result, people were asked to avoid crowding, and direct contact between people was significantly reduced. To relieve the stress caused by these changes, expectations of forests and green spaces became more fabulous than ever [21,22], not only in Europe, where lockdowns were implemented around the spring and summer of 2020 [23,24,25], but also in Japan, where the number of people affected was relatively low [26]. Studies have been conducted to assess the well-known effects of green spaces, such as trees seen through windows (e.g., hospital or house windows), and greenery in houses and neighborhood gardens that can be accessed even in situations that prevent people from going out unnecessarily, on the mental and physical health. Furthermore, some studies have reported that increasing the degree of greenery indoors [27], watching forest videos daily [28], touching wood [29], listening to the sounds of nature [30], looking at flowers [31], experiencing forest scenery [32] and sunlight through the foliage [33], and using virtual reality (VR) for watching natural scenery [29,30,31] can reduce stress.

Participating in the environment has been a key point in previous VR-based studies [34,35,36], and head-mounted displays (HMDs) have been used to present high-quality images for improving immersion. However, some people, such as people who are older and who have dementia and children with attention-deficit hyperactivity disorder, do not feel comfortable wearing HMDs. Under such conditions, in any case, most VR users are alone in their experience of nature, for now. They cannot share the natural environment and their experiences with others, which does not satisfy the fundamental human desire to share good experiences with other people. Therefore, if there is an environment or facility where people can share their experiences while solving the problem of wearing an HMD, it will be possible to have an experience that solves the above issues to a large extent. In addition, if such an artificial forest environment and facilities are available, even after the coronavirus pandemic, urban residents who have become physically and psychologically distant from forests in their daily lives can have a more carefree experience of the forest environment than before, which is expected to increase interest in and connection with real nature. The interest in forests nurtured in this way will encourage people to visit actual forests and make more active use of them. Furthermore, if forests are seen again as an integral part of people’s daily lives, it will lead to the proper management of Japan’s forests, which account for 67% of the country’s land area.

In this study, we constructed a digital forest bathing environment that reproduces a forest indoors by selecting and extracting environmental elements of a forest-related to sight, hearing, and smell. In addition, we investigated the physiological and psychological restorative effects of this digital forest bathing environment. The relative degree of the psychological restorative effect of the digital forest bathing environment and the characteristics of the environment that brought about the effect are examined by comparing these with corresponding measures of actual forest and urban environments.

## 2. Materials and Methods

### 2.1. Research Design

**Figure 1:**
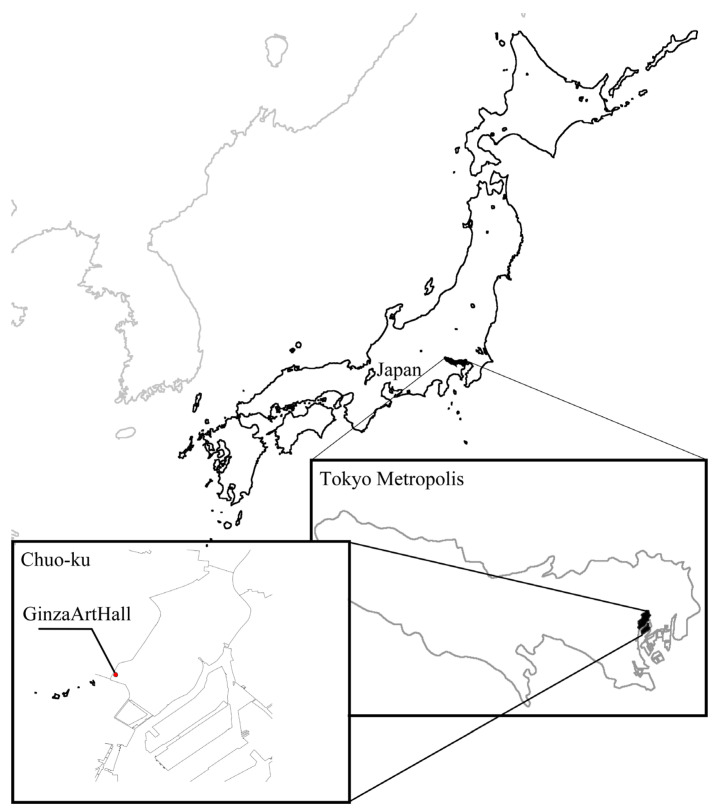
Location map of the experimental site.

The experiment was conducted over two days, 9 and 10 February 2020, at the Ginza Art Hall (hereafter referred to as art gallery) in the Ginza district of Chuo-ku, Tokyo, which was rented out. During the survey period, a state of emergency was declared for the entire Tokyo metropolitan area owing to the COVID-19 pandemic. Therefore, in line with the “Tokyo Metropolitan Government Guidelines for the Prevention of the Spread of Infectious Diseases,” we ventilated the experimental and waiting room; disinfected the seats with alcohol, as needed; and asked the subjects to wear masks (Figure 1). All subjects were not vaccinated before the study. In addition, a test kit was sent to the subject’s home in advance to check for COVID-19 infection just before the experiment. Although this study, similar to previous studies [7,8], is a case study and a type of study without a control, the experimental protocol was determined by referring to the literature [2,4,5,7,8,10] and by considering the burden on the subjects.

### 2.2. Participants

Participants were recruited from the public, regardless of age or gender, by publicizing the outline of this experiment on Facebook. Among the 60 applicants, 30 were selected (10 each in their 20s to 40s; all participants were Japanese) after confirming the presence of no pre-existing medical conditions. During recruitment, we attempted to enroll an equal number of men and women. On the day of the experiment, 25 people actually participated in the experiment (Table 1; mean age ± standard deviation (SD): all, 36.1 ± 8.13 years; male, 37.9 ± 9.97 years; female, 34.5 ± 8.23 years). In terms of age, we chose participants in their 20s, 30s, and 40s who are busy working and generally have few opportunities to experience forests and nature.

This study was conducted in accordance with the guidelines of the Declaration of Helsinki. In addition, it was approved by the Institutional Ethics Committee of the Forestry and Forest Products Research Institute, Japan (approval number: 031803, 2 December 2020).

### 2.3. Environment of Digital Forest Shinrin-Yoku

**Figure 2:**
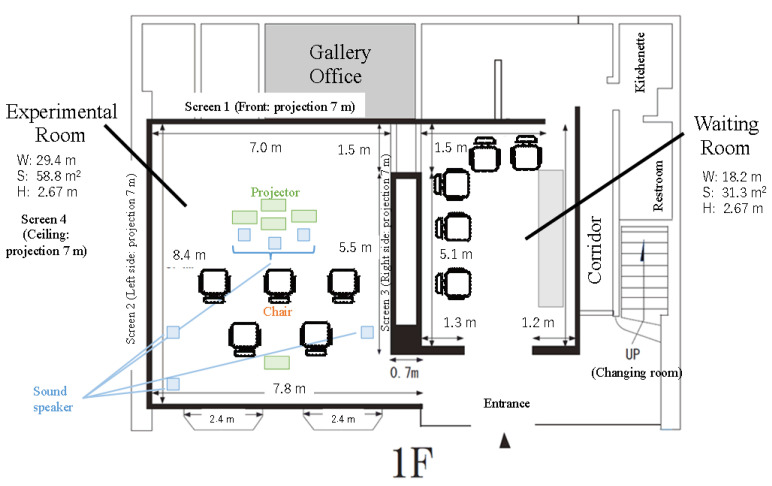
Layout of the experimental and waiting rooms.

We prepared two rooms for the experiment: a waiting room and an experimental room (Figure 2). In the anteroom (31.3 m^2^), we explained the experiment to the subjects; requested informed consent, after which they filled in the consent form; and conducted a psychological survey before the digital forest bathing experience. The experimental room (58.8 m^2^) was set up next to the waiting room and was designed to incorporate elements of the forest environment, focusing on sight, hearing, and smell to create an environment for digital forest bathing. Five projectors (FP-Z5000, FUJIFILM Holdings Corp., Tokyo, Japan) were used to project video images taken in Urahoro-cho, Hokkaido, on the three south–northwest walls and ceiling of the experimental room. Simultaneously, speakers (ICS-15, ONKYO Home Entertainment Corp., Osaka, Japan, one being a woofer) installed at six locations in the room emitted environmental sounds linked to the video images. The environmental sounds were recorded by a sound-collecting microphone at the same time that the video was taken (Insta360 ONE R, Shenzhen Arashi Vision Co., Ltd., Shenzhen, China). In addition, to reconstruct the forest environment of Urahoro-cho to the extent technically feasible, the essential oil of *Abies sachalinensis*, the main tree species in Urahoro-cho, was put into a diffuser (Orb, AT-AROMA Co., Ltd., Tokyo, Japan) and installed in the experimental room to ensure that the subjects could have an olfactory experience linked to the video. To avoid congestion during the experiment, the number of participating subjects was limited to a maximum of five per session, and the subjects sat facing the west wall, with some distance between them to experience digital forest bathing (Figure 2).

### 2.4. Physiological Indicators

We used seven indicators to evaluate the physiological effects of the digital forest bathing environment on restorativeness. The indicators were classified into those to be compared before and after the experience and those to be measured continuously during the experience. Specifically, four types of physiological indices were assessed before and after the experience of the digital forest bathing environment, and three physiological indices were assessed every s during the experience.

#### 2.4.1. Physiological Indices Measured before and after the Experience

**Figure 3:**
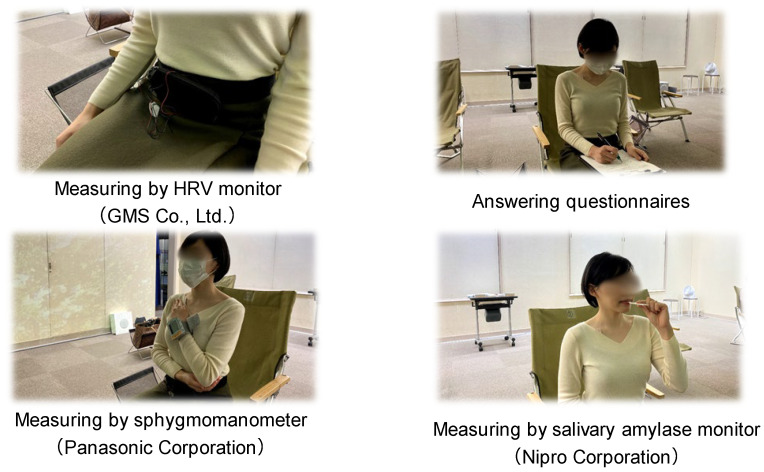
Measurement of each physiological indicator. Note: For the measurement of HRV, the sensor was attached to the body before the experiment, and the data logger was continuously fixed at the waist position for recording during the experiment. To measure blood pressure and pulse rate, a small arm-wrapped sphygmomanometer was used and fixed at the level of the heart each time during the measurements. Salivary amylase was also collected using a special regent. For the questionnaire survey, the experimental room was lit so that the participants could see their hands when answering the questions.

Before and after experiencing the digital forest bathing environment, we measured participants’ maximum and minimum blood pressure, pulse rate [37,38,39,40], and salivary amylase activity [34,41]. For maximum and minimum blood pressure as well as pulse rate, five arm-wrap sphygmomanometers (HEM-6022, Omron Corp., Kyoto, Japan, etc.) were prepared and attached to the arms of each subject for measurement. During measurement, the subjects were seated, with their arm fixed at the level of the heart. Two measurements were taken at each time point, and the average value was used for analysis (Figure 3). For salivary amylase activity, four salivary amylase monitors (59-014, Nipro Corp., Osaka, Japan) were used to measure and record changes in the subjects’ condition before and after the experience (Figure 3).

#### 2.4.2. Physiological Indices Measured Continuously during the Experience

To investigate the state of sympathetic and parasympathetic nervous activity [42,43,44] and heart rate [45] throughout the experiment, an adhesive sensor and an active tracer (AC-301A, GMS Co., Ltd., Cheonan, Korea) were attached to each subject. The active tracer can measure the heart rate every millisecond. Thus, the sympathetic nerve activity, parasympathetic nerve activity, and heart rate while exposed to the digital forest bathing environment were evaluated by assessing heart rate fluctuation (Figure 3).

### 2.5. Psychological Indicators and Environmental Appraisal

For evaluating the psychological effects of the digital forest bathing environment and the characteristics of the environment that can recover our body and mind (restorative traits), we used the following questionnaires, referring to previous studies [5,10,37,38,42,46,47,48,49,50].

#### 2.5.1. Profile of Mood States (POMS; Mood)

POMS is a famous psychological questionnaire that assesses mood states using six subscales: Tension–Anxiety, Depression, Anger–Hostility, Vigor, Fatigue, and Confusion. It was developed by McNair et al. [46] and was adapted into a Japanese version by Yokoyama et al. [47]. It has been used in many related studies [48,49,50] and is highly reliable. It is also easy to compare with the results of other studies.

#### 2.5.2. Positive and Negative Affect Schedule (PANAS; Affect)

PANAS is a psychological index that investigates the affect from two subscales, “Positive Affect” and “Negative Affect.” It was developed by Watson et al. [51,52] and was adapted to a Japanese version by Sato and Yasuda [53]. It has been used in studies on forest bathing by Takayama et al. [49] and Bielinis et al. [50].

#### 2.5.3. Restorative Outcome Scale (ROS; Subjective Restorativeness)

ROS is a psychometric measure of subjective restorativeness developed by Korpera et al. [54,55] and has no subscales. It has been used by Ojala et al. [56] and other researchers [49,50] and has been used in on-site studies on forest bathing. The Japanese version of this scale was prepared by Fujisawa and Takayama [57] for surveying.

#### 2.5.4. Perceived Restorativeness Scale (PRS; Appraisal for Restorative Trait in an Environment)

PRS was developed by Hartig et al. [58] based on the Attention Restoration Theory (ART) by Kaplan and Kaplan [59]. PRS can investigate the restorative traits of digital forest bathing environments by comparing them with the traits of other settings [60]. It consists of seven subscales: being away, fascination, coherence, scope, compatibility, preference, and familiarity. In this study, only indices related to restorative traits were used. Therefore, only five subscales related to ART (being away, fascination, coherence, scope, and compatibility) were analyzed.

### 2.6. Experimental Procedure

**Figure 4:**
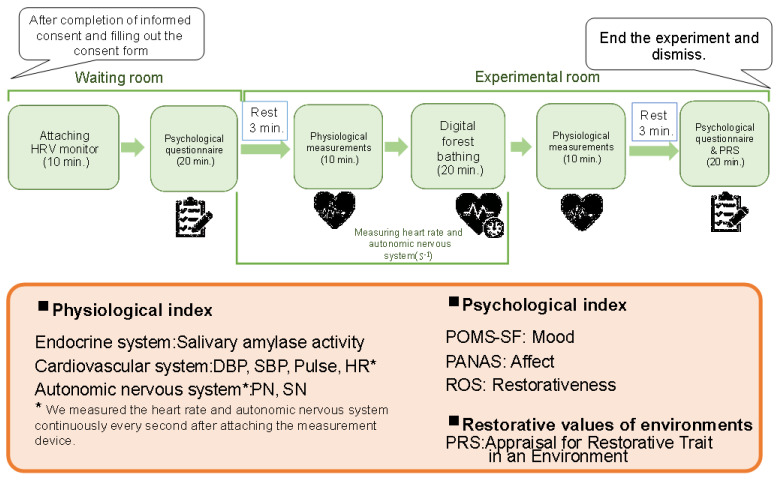
Experimental protocols and indicators.

The experiment in the digital forest bathing environment was conducted according to the procedure shown in Figure 4. It was divided into three sessions: (i) 11:30–13:30, (ii) 14:00–16:00, and (iii) 16:30–18:30 on both days (six sessions in total). To prevent new coronavirus infection, the maximum number of subjects participating in each session was limited to five, and adequate distance was ensured between subjects in the waiting and experimental rooms. Of the 30 candidates, 25 participated in the experiment (Table 1) (12 male and 13 female participants).

The subjects who participated in the experiment first registered at the reception desk in the building after arriving at the Ginza Art Hall. Then, in the waiting room, they were introduced to the experimental staff, the purpose of the experiment was explained, and they signed an informed consent form.

After the subjects signed the consent form, the staff directed them to a changing room where adhesive sensors and active tracers (sympathetic nervous system activity, parasympathetic nervous system activity, and heart rate) were attached to the body using a belt.

After the subjects returned to the waiting room after installing the tracers and sensors, the active tracer was switched on, and participants were asked to answer each questionnaire (POMS, PANAS, and ROS) as a part of psychological survey before the experience.

Subsequently, the subjects were immediately moved to the experimental room next to the waiting room. After sitting on the outdoor chair, the subjects were allowed to rest (3 min). After resting, their preliminary physiological survey parameters (maximum blood pressure, minimum blood pressure, pulse rate, and salivary amylase activity) were measured. Values corresponding to these measures were recorded on a sheet by the experimental staff (10 min).

**Figure 5:**
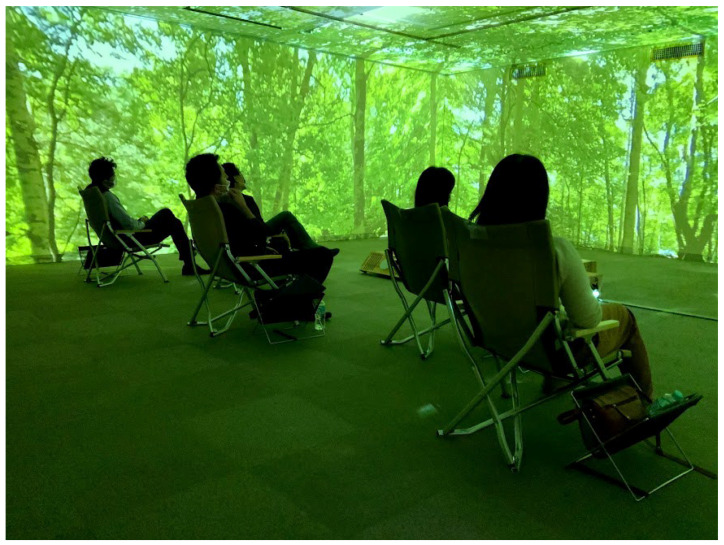
The experiment in progress.

After completing the physiological measurements, the subjects were allowed to experience a digital forest bath for 20 min (Figure 5).

Post-experience physiological measurements (maximum blood pressure, minimum blood pressure, pulse rate, and salivary amylase activity) were obtained at the end of the digital forest bath (10 min).

In addition, for psychological measurements after the experience, subjects were asked to answer POMS, PANAS, and ROS questionnaires. Finally, to investigate the restorative traits of the digital forest bathing environment, we asked the subjects to complete the PRS questionnaire.

Subsequently, the subjects who completed all the experimental processes received their rewards and went home.

### 2.7. Data Analysis

After organizing the acquired data, the physiological indices (maximum blood pressure, minimum blood pressure, pulse rate, and salivary amylase activity) measured before and after the experience were organized by index, and the mean values before and after the experience were compared and tested. Standardized values for each device were used in the analysis for salivary amylase activity because individual differences were noted among measurement devices.

The physiological indices (heart rate and heart rate variability) measured continuously by the active tracer were analyzed as follows. (1) Heart rate was measured every millisecond and cut out every 30 s. (2) The data for 90 s after the start of resting was deleted, and the remaining 90 s was used for analysis as control. The data for 90 s after the start of digital forest bathing was deleted, and the data for the remaining 18 min and 30 s was used for the analysis (Figure 4). (3) Frequency analysis was performed on the heart rate at 30-s intervals using MemCalc/Win (GMS Co., Ltd., Tokyo, Japan) to obtain LF (Low Frequency) and HF (High Frequency). The standardized LF/HF value for each subject was used as an index of sympathetic nerve activity, and the similarly standardized HF was used as an index of parasympathetic nerve activity. 

In contrast, the psychological indices measured before and after the experience using POMS and PANAS were aggregated and organized according to the subscales that constituted each index. Then, the mean values before and after the experience were compared. For the POMS, T-scores were calculated and used in the analysis; for ROS, they were aggregated as they were for comparison and testing, since the scale does not have subscales. Next, the results of POMS, PANAS, and ROS were compared with those obtained by the authors in their previous study [49], which were measured in actual urban and forest environments. In addition, the PRS data were compared with those obtained in a previous study [61] investigating the recovery characteristics of urban and forest environments. Both studies [49,61] investigated the psychological effects of a short stay in forest and urban environments on 45 subjects in their 20s as well as assessed the recovery characteristics of both environments. Excel 2019 (Microsoft Corp., Washington, DC, USA) was used to organize data, and SPSS Statistics 28 (IBM Corp., New York, NY, USA) was used for comparison and testing.

## 3. Results

### 3.1. Physical Environment

Temperature, relative humidity, and average illuminance in the experimental and waiting rooms are shown in Table 2. The average illuminance in the experimental room is described at rest and when answering the questionnaires as well as when experiencing the digital forest bathing environment.

### 3.2. Physiological Effects

There were no significant differences in diastolic blood pressure, systolic blood pressure, pulse rate, and salivary amylase activity before and after experiencing the digital forest bathing environment (Table 3). Heart rate was significantly (*p* < 0.01) lower during the experimental state than during the resting state (Table 4).

### 3.3. Psychological Effects

#### 3.3.1. Before and after Comparison

Data from each psychological questionnaire were organized and analyzed for differences in psychological states before and after the experience of digital forest bathing. As for the mood states (measured using POMS), significant (*p* < 0.01) decreases were observed for “Tension–Anxiety,” “Anger–Hostility,” “Fatigue,” and “Confusion” after the experience. There was also a downward trend (marginally significant, *p* < 0.1) for “Depression-depression,” but no characteristic trend was observed for “liveliness.” As for emotions (measured using PANAS), negative emotions were significantly (*p* < 0.01) lower after the experience. In contrast, there was no characteristic change in positive emotions. The feeling of recovery (measured using ROS) increased significantly (*p* < 0.01) after the experience (Table 5).

#### 3.3.2. Comparison between Different Environments

As the current experiment had no control, we tried to refer to the results of previous approximate studies to examine the results of digital forest bathing from multiple perspectives. Table 6 rearranges the data from studies in past literature [49]. It shows the results of psychological studies conducted in both real forest and urban environments. When comparing the actual urban and forest environments [49], no significant differences were noted for five of the six subscales of POMS in the pre-experience mood state. Only “Vigor” was significantly (*p* < 0.01) higher in the digital forest bathing environment than in the urban and forest environments (Table 7 and Table 8).

The post-experience comparison showed that the digital forest bathing environment and the forest environment were associated with significantly (*p* < 0.01) lower “Tension–anxiety” and “Fatigue” than the urban environment. In addition, the post-experience “Fatigue” was significantly (*p* < 0.05) lower after experiencing the digital forest bathing environment than after experiencing the actual forest environment (no other subscale showed a significant difference between these two environments in the post-experience comparison). In addition, “Anger–Hostility” was significantly (*p* < 0.01) lower in the digital forest bathing environment than in the urban environment. For post-experience “Depression” and “Confusion,” no significant differences were identified between the digital forest bathing environment and the other environments. After the experience, “Vigor” differed significantly between the digital forest bathing environment and urban environment (*p* < 0.01) and between the forest environment and urban environment. However, this difference in scores was non-existent before the experience (Table 7 and Table 8).

Furthermore, the “Negative affect” before the experience (measured using PANAS) did not differ significantly between the environments; however, it differed significantly after the experience (*p* < 0.01) and was lower in the digital forest bathing and forest environments than in the urban environment. In contrast, before the experience, the “Positive affect” score of the digital forest bathing environment was significantly (*p* < 0.01) higher than that of the urban and forest environments. After the experience, the score of the digital forest bathing environment was significantly (*p* < 0.05) higher than that of the urban environment [49] (Table 9 and Table 10).

Finally, in terms of the feeling of recovery (measured using ROS), no significant differences were identified among the environments before the experience. However, after the experience, scores of the digital forest bathing environment and the forest environment were significantly (*p* < 0.01) higher than those of the urban environment, with no significant difference between the digital forest bathing environment and the actual forest environment (Table 11 and Table 12).

### 3.4. Comparison between Different Environments of Restorative Trait in an Environment

For the same reason as for psychological effects, to investigate the preparedness and recovery characteristics of the digital forest bathing environment, the digital forest bathing and urban and forest environments were compared using the responses to the Perceived Restorativeness Scale (PRS) questionnaire and the PRS data used in previous studies [61] (Table 13 and Table 14).

For the “Being away” subscale, the scores in digital forest bathing environment and forest environment were significantly (*p* < 0.01) higher than those in the urban environment. Still, there was no significant difference between the scores of digital forest bathing environment and forest environment. For the “Fascination” subscale, there was no significant difference between the digital forest bathing environment scores and the other environment scores. Furthermore, for “Coherence,” the scores of the digital forest bathing environment tended to be lower than those of the actual forest (marginally significant; *p* < 0.1). In contrast, the scores of “Scope” tended to be higher in the digital forest bathing environment than in the urban environment (marginally significant; *p* < 0.1). As for “Compatibility” between the environment and behavior, the score of digital forest bathing environment was significantly (*p* < 0.01) higher than of the urban environment (Table 14).

## 4. Discussion

### 4.1. Physiological Effects

Many previous studies have discussed the physiological restorative effects of actual forest bathing. In addition, the physiological relaxation effects of simulated forest bathing experiences in V.R. environments [34,35,36] have been confirmed. However, in the present study, the indices before and after experiencing the digital forest bathing environment had no significant differences (Table 3). This was because the subjects were allowed to relax during the digital forest bathing experience but were asked not to fall asleep. However, from the results of the post-experimental interviews, approximately half the subjects reported that they felt sleepy because they were too relaxed. Therefore, perhaps it was assumed that the state of enduring not to fall asleep during the experiment became stressful, and the physiological effects that should have been obtained were offset as a whole.

In contrast, information on heart rate fluctuations calculated every s revealed that parasympathetic nerve activity significantly increased and that heart rate significantly decreased while experiencing the digital forest bathing environment than while in the resting state before the experience (Table 4). This result is similar to that reported in previous studies that showed that parasympathetic nerve activity increased [42,43,44] and that heart rate decreased [42,43] during actual forest bathing and plant observation. Therefore, this result might mean that the subjects were physiologically relaxed while experiencing the digital forest bathing environment.

### 4.2. Psychological Effects

Comparing the results of the test for Before (before the experience) for each indicator in Table 8, Table 10 and Table 12, the scores were quite close to each other without any significant differences, except for some (Vigor in POMS and Positive affect in PANAS).

Therefore, assuming that the subject groups were homogeneous to a considerable extent, to discuss the results of this study in a more multifaceted perspective, we thought that we could discuss the psychological effects and restorative traits of digital forest bathing compared with the actual forest and urban environments.

First, from the comparison of the mood states (Table 5), the mood state improved after experiencing the digital forest bathing environment in all subscales, except “Vigor.” Furthermore, compared with urban and forest environments (Table 6), the digital forest bathing environment significantly improved the mood state, indicating that the digital forest bathing environment has similar effects to the actual forest environment (Table 7 and Table 8).

Regarding the affects, “Negative affect” was significantly lower after experiencing the digital forest bathing environment (Table 5). Compared with other settings (Table 6), the digital forest bathing environment had a significantly lower negative affect after the experience than the urban environment, showing a similar trend to the actual forest environment (Table 9 and Table 10). In contrast, there was a slight decrease in “Positive affect” after the experience; however, no significant difference could be confirmed before the experience (Table 5). The digital forest bathing environment scores were significantly higher than the urban environment scores pre- and post-experience (Table 5, Table 6 and Table 10). Still, they were significantly higher than the urban and forest environment scores in the pre-experience comparison (Table 10). In addition, the fact that the scores in the forest and urban environments increased (forest = *p* < 0.05; city (urban) = n.s.) after the experience (Table 6) and those in the digital forest bathing environment decreased (n.s.; Table 5) suggested that participants’ expectations of the digital forest bathing environment were relatively high before the experience. After actually experiencing it, their feelings were somewhat different. In other words, a positive affect was unknown. Thus, the tendency of a negative affect might be similar to that of the actual forest environment, although the positive affect was still unclear.

In addition, the sense of restorativeness increased significantly after experiencing the digital forest bathing environment (Table 5). The urban and forest environments did not differ significantly with regard to the sense of restorativeness before the experience, but the forest environment was associated with a significantly higher sense of restorativeness after the experience. This parameter did not differ significantly before the experience in the urban and forest environments, but it was significantly higher in the forest environment after the experience. As this tendency was similar to that of the forest environment, the sense of recovery may be similar to that of the actual forest environment (Table 11 and Table 12).

From the above results, it was confirmed that the digital forest bathing environment decreased negative psychological factors, such as “Tension–Anxiety,” “Depression,” “Anger–Hostility,” “Fatigue,” “Confusion,” and “Negative affect,” and increased the sense of restorativeness, similar to the effects of an actual forest environment.

### 4.3. Restorative Trait of an Environment

The factors contributing to the physiological and psychological effects of the digital forest bathing environment on the subjects were considered from the perspective of the restorative properties of the environment. As shown in Table 13 and Table 14, compared with the urban environment, the digital forest bathing environment was associated with significantly higher (or significantly higher tendency) scores for the “Being away,” “Scope,” and “Compatibility” subscales among the five subscales related to restorative traits. In addition, the scores of each indicator were similar to the scores of the forest environment, and the only difference (marginally significant) between them was for “Coherence.” As described by Shibata et al., “Coherence” was treated as an item to measure the degree of coherence among the elements constituting the environment [60]. However, in the digital forest bathing environment, the stimuli of forest scenery and sound were projected every 5 min so that the subjects were not bored in the closed space, and the stimuli that constituted the environment were limited and different from the “coherent image of the forest” that they had acquired through their past forest experiences. However, the digital forest bathing environment might be quite similar to the forest environment in terms of restorative traits. Owing to this commonality of restorative traits, the physiological and psychological restorative effects on the subjects were possibly similar to those of the actual forest environment.

## 5. Conclusions

In this study, a digital forest bathing environment was constructed in a closed indoor space, and men and women in their twenties to forties were enrolled to experience it. The physiological and psychological effects of the subjects were investigated by comparing the measurement results before and after the experience and by continuous measurement. In addition, cross-sectional comparison of the psychological effects and the restorative traits of the environment compared with other settings was conducted by comparing data on urban and forest environments obtained in the past studies. The results revealed a certain degree of physiological and psychological restorative effects following a short experience of digital forest bathing. In addition, the psychological effects were similar to those obtained in an actual forest environment. In addition, the restorative traits of the digital forest bathing environment were similar to those of the real forest environment.

This time, we experimented under a declared state of emergency caused by COVID-19. As we already discussed, if digital forest bathing can have an effect similar to that of a real forest, then it can be used as a new device to reduce people’s mental and physical stress, even in the event of another pandemic caused by COVID-19 or a new infectious disease. In this way, digital forest bathing can be used as a new device to reduce mental and physical stress. For example, even when people are restricted from going out due to lockdowns, they can enjoy a safe and comfortable forest bathing experiences with their family and friends if they can create a digital forest bathing environment in their living room.

Here are some of the research questions. First, we planned to enroll 30 subjects in the experiment; however, because of COVID-19, only 25 subjects participated. To avoid such a situation, it is necessary to take the following measures: (1) ensure that the experiment site is safe and has sufficient COVID-19 countermeasures (and proactively announce such countermeasures), (2) contact the candidates the day before the experiment to maintain their interest, and (3) ensure spare subjects in advance in case of sudden nonparticipation. Additionally, it should be noted that the use of Facebook as a method of gathering participants limited the number of people who wanted to participate to those who use Facebook and that the ease of applying for participation may have resulted in a large number of absences on the day of the experiment among those who planned to participate.

In addition, it seems necessary to provide sufficient consideration to the “Vigor” subscale of POMS. Furthermore, it was found that positive subscales such as “Vigor” of POMS and “Positive affect” of PANAS were significantly higher than in other environments even before experiencing the digital forest bathing. We cannot deny the possibility that the high expectation of the participants for experiencing the digital forest bathing in an art gallery in Ginza (one of the most luxurious shopping areas in Tokyo) as the experimental setting was reflected in the scores of the positive subscales before the experience. The reason why there was no significant difference in the physiological indices before and after the digital forest bathing experience, unlike previous studies, may be related to the intensity of the digital forest bathing stimulus, the exposure time, and the appropriateness of the indexes. Therefore, it is necessary to carefully examine this issue again in the future. In this study, we used data on urban and forest environments collected by the authors in a previous survey to compare the digital forest bathing environment. However, a more accurate analysis would have been possible if similar subjects were enrolled. We hope that this study will help design future research projects.

Finally, this article is a case study, so the findings are limited. As we discussed so far, there is a possibility that the digital forest bathing environment has restorative traits similar to those of an actual forest environment and that restorative effects similar to those of a forest environment can be obtained. Moreover, as it is possible to respond to various demands by changing the material that becomes a stimulus, it can be said that the future applicability and development potential is very high. However, it should be noted that the individual materials that make up a digital forest environment are recorded for the natural environment and already fixed while they are themselves replaceable materials. In other words, it is better to think of it as something different from the highly coincidental experience that is experienced when visiting an actual natural environment owing to a combination of factors such as nature, season, time of day, and weather. Therefore, we recommend that people in the city who are interested in experiencing nature but are unsure on how to do so, such as businessmen or patients in hospitals or nursing homes, should be exposed to nature so that they can recover from their daily stress and recall their past experiences. Thus, it would be desirable to develop a similar tool to help people recover from daily stress, to remember past experiences, and to become happier or as a gateway to the forest.

## Figures and Tables

**Table 1:** Characteristics and age structure of subjects.

Age of Subjects	Male	Female	Total
20s	2	6	8
30s	5	3	8
40s	5	4	9
Total	12	13	25

**Table 2:** Temperature, relative humidity, and illumination in experimental and waiting rooms.

		Temperature (°C)	Relative Humidity (%)	Illuminance (lx)
Experimental room	Baseline	27.29 ± 2.11	11.15 ± 2.54	49.74 ± 4.01
	During Shinrin-yoku	27.52 ± 1.22	11.31 ± 2.66	23.33 ± 0.505
	During Measurement *	26.36 ± 0.630	12.43 ± 2.70	71.21 ± 3.46
Waiting room	Baseline	20.51 ± 2.53	18.39 ± 3.23	1453.47 ± 181.13

* Responding to the PRS questionnaire.

**Table 3:** Comparison of physiological indicators (before and after the experience).

Physiological Index	Before	After	*p*	d
Systolic blood pressure (mmHg)	118.48 ± 14.40	119.22 ± 16.01	0.710	0.049
Diastolic blood pressure (mmHg)	80.24 ± 9.76	81.76 ± 10.52	0.182	0.150
Pulse Rate (bpm)	70.04 ± 8.35	68.82 ± 7.62	0.188	0.153
Amylase	−0.059 ± 0.970	0.059 ± 0.984	0.685	0.122

Paired *t*-test, d: effect size (Cohen’s d), *n* = 25.

**Table 4:** Comparison of physiological indices (control and experimental values).

Physiological Index	Experimental	Control	*p*	d
LF/HF	0.135 ± 0.286	−0.031 ± 0.469	0.165	0.427
HF	0.199 ± 0.428	−0.161 ± 0.741	0.014 *	0.595
Heart Rate (bpm)	70.67 ± 8.66	74.85 ± 9.17	<0.001 **	0.468

LF/HF (Low Frequency/Hi Frequency) and HF (Hi Frequency) mean the sympathetic nerve activity and parasympathetic nerve activity in each: Paired *t*-test; *: *p* < 0.05, **: *p* < 0.01, d: effect size (Cohen’s d), *n* = 25.

**Table 5:** Comparison of before and after digital forest bathing experience.

Physiological Index		Before	After	*p*	d
POMS	T-A	43.36 ± 8.59	36.04 ± 3.60	<0.001 **	1.11
	D	44.52 ± 7.01	41.76 ± 5.33	0.042 *	0.443
	A-H	41.12 ± 4.75	37.56 ± 1.76	<0.001 **	0.993
	V	49.28 ± 13.01	47.72 ± 15.10	0.560	0.111
	F	43.12 ± 8.55	37.16 ± 2.94	<0.001 **	0.933
	C	47.92 ± 8.28	43.16 ± 6.53	<0.001 **	0.639
PANAS	Negative	12.32 ± 5.91	9.4 ± 2.71	0.002 **	0.635
	Positive	31.52 ± 12.58	28.6 ± 14.57	0.171	0.215
ROS		25.88 ± 6.50	30.96 ± 5.97	<0.001 **	0.814

Paired *t*-test; *: *p* < 0.05, **: *p* < 0.01, d: effect size (Cohen’s d), *n* = 25.

**Table 6:** Review of previous studies comparing digital forest bathing.

Location		Forest				City			
Physiological Index		Before	After	*p*	d	Before	After	*p*	d
POMS	T-A	43.15 ± 7.86	39.15 ± 5.84	<0.001 **	0.578	42.39 ± 8.04	43.98 ± 7.41	0.199	0.205
	D	44.00 ± 5.49	42.37 ± 4.86	0.006 **	0.314	43.52 ± 6.24	43.63 ± 5.40	0.851	0.019
	A-H	41.89 ± 7.30	39.63 ± 4.42	0.026 *	0.375	40.33 ± 6.29	40.96 ± 4.73	0.414	0.113
	V	42.78 ± 10.16 *	45.15 ± 9.41	0.115	0.242	41.41 ± 9.08	36.35 ± 8.43	<0.001 **	0.578
	F	43.83 ± 9.11	42.7 ± 9.06	0.322	0.124	44.87 ± 9.26	49.54 ± 10.00	<0.001 **	0.485
	C	44.13 ± 8.19	40.94 ± 5.72	0.009 **	0.453	43.87 ± 6.89	45.61 ± 7.69	0.025 *	0.238
PANAS	Negative	11.96 ± 6.08	11.76 ± 5.98	0.854	0.032	14.20 ± 8.26	16.26 ± 8.14	0.078	0.252
	Positive	22.00 ± 9.35	23.93 ± 9.77	0.04 *	0.202	20.59 ± 9.40	21.39 ± 10.07	0.553	0.083
ROS		26.17 ± 5.56	29.6 ± 6.39	<0.001 **	0.573	25.13 ± 6.27	21.13 ± 8.73	<0.001 **	0.527

Paired *t*-test; *: *p* < 0.05, **: *p* < 0.01, d: effect size (Cohen’s d), *n* = 45 (Forest and City). Note: Table 6 was created by quoting the POMS, PANAS, and ROS data obtained by Takayama et al. [49]; Takayama et al. (2014) conducted on-site experiments (10–12 subjects) in four municipalities in Japan to compare and examine some psychological effects of a short stay in the forest (one deciduous forest, one coniferous forest, and two mixed coniferous and broadleaf forests) and city (all four locations: the central area representative of each municipality) environments. The numerical values in Table 6 show the averages and SDs before (Before) and after (After) the stay at the four locations (both forest and city), the results of the *t*-test for the forest and city means (*p*), and the effect size (d) at the time of the test. Although there are differences, such as that (1) Takayama et al. (2014) used real forest and urban environments as the target sites; (2) the subjects were mainly in their 20s; and (3) with the duration of stay being 30 min, by conducting experiments using the same index, it is possible to compare and discuss the effects of digital forest bathing to those of actual forest and urban environments.

**Table 7:** Comparative analysis of POMS in three settings (two-way analysis of variance).

Psychological Index		Main Effect						Interaction		
		Location (Digital Forest vs. Forest vs. City)			Time (before vs. after)			Location × Time		
		F	*p*	η^2^	F	*p*	η^2^	F	*p*	η^2^
POMS	T-A	4.25	0.015 *	0.032	11.21	0.001 **	0.042	7.20	0.001 **	0.054
	D	0.150	0.861	0.001	3.49	0.063	0.014	1.18	0.309	0.010
	A-H	1.26	0.286	0.010	5.60	0.019 *	0.023	2.97	0.053	0.024
	V	14.79	<0.001 **	0.108	1.02	0.315	0.004	3.04	0.050 *	0.022
	F	11.36	<0.001 **	0.084	0.457	0.500	0.002	6.38	0.002 **	0.047
	C	3.60	0.029 *	0.028	4.51	0.035 *	0.018	4.33	0.014 *	0.034

Two-way analysis of variance: *: *p* < 0.05, **: *p* < 0.01, η^2^: effect size, *n* = 25 (digital forest), *n* = 45 (forest and city).

**Table 8:** Analysis results of POMS in three settings (multiple comparisons).

Time	Location		Digital Forest vs. Forest		Digital Forest vs. City		Forest vs. City	
	Index		*p*	d	*p*	d	*p*	d
Before	POMS	T-A	1.00	0.025	1.00	0.116	1.00	0.096
		D	1.00	0.083	1.00	0.151	1.00	0.081
		A-H	1.00	0.125	1.00	0.142	0.477	0.230
		V	0.037 *	0.557	0.008 **	0.701	1.00	0.142
		F	1.00	0.080	1.00	0.196	1.00	0.114
		C	0.105	0.460	0.073	0.532	1.00	0.034
After	POMS	T-A	0.242	0.642	<0.001 **	1.36	0.003 **	0.723
		D	1.00	0.119	0.547	0.349	0.831	0.245
		A-H	0.369	0.616	0.035 *	0.953	0.697	0.290
		V	0.960	0.204	<0.001 **	0.930	<0.001 **	0.986
		F	0.035 *	0.822	<0.001 **	1.68	<0.001 **	0.718
		C	0.642	0.363	0.515	0.343	0.005 **	0.689

Bonferroni correction: *: *p* < 0.05, **: *p* < 0.01, d: effect size, *n* = 25 (digital forest), *n* = 45 (forest and city).

**Table 9:** Comparative analysis of PANAS in three settings (two-way analysis of variance).

Psychological Index		Main Effect						Interaction		
		Location (Digital Forest vs. Forest vs. City)			Time (before vs. after)			Location × Time		
		F	*p*	η^2^	F	*p*	η^2^	F	*p*	η^2^
PANAS	Negative	9.11	<0.001 **	0.070	0.147	0.702	<0.001	2.29	0.104	0.018
	Positive	12.42	<0.001 **	0.095	0.002	0.966	<0.001	0.883	0.415	0.007

Two-way analysis of variance: **: *p* < 0.01, η^2^: effect size, *n* = 25 (digital forest), *n* = 45 (forest and city).

**Table 10:** Analysis results of PANAS in three settings (multiple comparisons).

Time	Location		Digital Forest vs. Forest		Digital Forest vs. City		Forest vs. City	
	Index		*p*	d	*p*	d	*p*	d
Before	PANAS	Negative	1.00	0.061	0.783	0.261	0.317	0.309
		Positive	<0.001 **	0.859	<0.001 **	0.985	1.00	0.151
After	PANAS	Negative	0.471	0.509	<0.001 **	1.13	0.004 **	0.630
		Positive	0.224	0.376	0.019 *	0.576	0.722	0.256

Bonferroni correction: *: *p* < 0.05, **: *p* < 0.01, d: effect size, *n* = 25 (digital forest), *n* = 45 (forest and city).

**Table 11:** Comparative analysis of ROS in three settings (two-way analysis of variance).

Psychological Index	Main Effect						Interaction		
	Location (Digital Forest vs. Forest vs. City)			Time (before vs. after)			Location × Time		
	F	*p*	η^2^	F	*p*	η^2^	F	*p*	η^2^
ROS	15.74	<0.001 **	0.109	2.76	0.098	0.010	10.48	<0.001 **	0.072

Two-way analysis of variance: **: *p* < 0.01, η^2^: effect size, *n* = 25 (digital forest), *n* = 45 (forest and city).

**Table 12:** Analysis results of ROS in three settings (multiple comparisons).

Time	Location	Digital Forest vs. Forest		Digital Forest vs. City		Forest vs. City	
	Index	*p*	d	*p*	d	*p*	d
Before	ROS	1.00	0.049	1.00	0.117	1.00	0.176
After	ROS	1.00	0.219	<0.001 **	1.31	<0.001 **	1.11

Bonferroni correction: **: *p* < 0.01, d: effect size, *n* = 25 (digital forest), *n* = 45 (forest and city).

**Table 13:** Results of comparative analysis by PRS in three settings (analysis of variance).

PRS	Digital Forest	Forest	City	Digital Forest vs. Forest vs. City		
				F	*p*	η^2^
Being away	35.44 ± 8.62	34.48 ± 12.24	19.07 ± 12.69	25.88	<0.001 **	0.305
Fascination	31.12 ± 9.53	31.96 ± 9.58	27.13 ± 9.50	3.34	0.039 *	0.054
Coherence	18.24 ± 8.07	22.39 ± 6.83	20.65 ± 8.06	2.48	0.088	0.040
Scope	22.64 ± 9.78	27.52 ± 9.00	17.39 ± 8.75	14.98	<0.001 **	0.202
Compatibility	29.48 ± 8.81	28.20 ± 7.83	23.65 ± 6.68	6.44	0.002 **	0.098

One-way ANOVA: *: *p* < 0.05, **: *p* < 0.01, η^2^: effect size, *n* = 25 (digital forest), *n* = 45 (forest and city).

**Table 14:** Comparison results by PRS in three settings (multiple comparisons).

PRS	Digital Forest vs. Forest		Digital Forest vs. City		Forest vs. City	
	*p*	d	*p*	d	*p*	d
Being away	1.00	0.091	<0.001 **	1.51	<0.001 **	1.24
Fascination	1.00	0.088	0.278	0.419	0.044 *	0.506
Coherence	0.086	0.555	0.601	0.299	0.793	0.233
Scope	0.093	0.519	0.062	0.566	<0.001 **	1.14
Compatibility	1.00	0.154	0.007 **	0.746	0.012 *	0.624

Bonferroni correction: *: *p* < 0.05, **: *p* < 0.01, d: effect size, *n* = 25 (digital forest), *n* = 45 (forest and city).
